# Role of microRNA/Epithelial-to-Mesenchymal Transition Axis in the Metastasis of Bladder Cancer

**DOI:** 10.3390/biom10081159

**Published:** 2020-08-07

**Authors:** Milad Ashrafizadeh, Kiavash Hushmandi, Mehrdad Hashemi, Mohammad Esmaeil Akbari, Peter Kubatka, Mehdi Raei, Lenka Koklesova, Md Shahinozzaman, Reza Mohammadinejad, Masoud Najafi, Gautam Sethi, Alan Prem Kumar, Ali Zarrabi

**Affiliations:** 1 Department of Basic Science, Faculty of Veterinary Medicine, University of Tabriz, Tabriz 5166616471, Iran; dvm.milad73@yahoo.com; 2 Department of Food Hygiene and Quality Control, Division of Epidemiology & Zoonoses, Faculty of Veterinary Medicine, University of Tehran, Tehran 1419963114, Iran; houshmandi.kia7@ut.ac.ir; 3 Department of Genetics, Faculty of advanced Science and Technology, Tehran Medical Sciences, Islamic Azad University, Tehran 1916893813, Iran; mhashemi@iautmu.ac.ir; 4 Cancer Research Center, Shahid Beheshti University of Medical Sciences, Tehran 1989934148, Iran; profmeakbari@gmail.com; 5 Department of Medical Biology and Division of Oncology—Biomedical Center Martin, Jessenius Faculty of Medicine, Comenius University in Bratislava, 03601 Martin, Slovakia; peter.kubatka@uniba.sk; 6 Health Research Center, Life Style Institute, Baqiyatallah University of Medical Sciences, Tehran 1435916471, Iran; Mehdi_r_d@yahoo.com; 7 Department of Obstetrics and Gynecology, Martin University Hospital and Jessenius Faculty of Medicine in Martin, Comenius University in Bratislava, 03601 Martin, Slovakia; koklesova.lenka@gmail.com; 8 Department of Nutrition and Food Science, University of Maryland, College Park, MD 20742, USA; mshahin81@gmail.com; 9 Pharmaceutics Research Center, Institute of Neuropharmacology, Kerman University of Medical Sciences, Kerman 55877577, Iran; r.mohammadinjead87@gmail.com; 10 Radiology and Nuclear Medicine Department, School of Paramedical Sciences, Kermanshah University of Medical Sciences, Kermanshah 6715847141, Iran; najafi_ma@yahoo.com; 11 Department of Pharmacology, Yong Loo Lin School of Medicine, National University of Singapore, Singapore 117600, Singapore; csiapk@nus.edu.sg; 12 Cancer Science Institute of Singapore, Centre for Translational Medicine, 14 Medical Drive, #11-01M, Singapore 117599, Singapore; 13 Sabanci University Nanotechnology Research and Application Center (SUNUM), Tuzla, Istanbul 34956, Turkey; 14 Center of Excellence for Functional Surfaces and Interfaces (EFSUN), Faculty of Engineering and Natural Sciences, Sabanci University, Tuzla, Istanbul 34956, Turkey

**Keywords:** bladder cancer, metastasis, epithelial-to-mesenchymal transition (EMT), microRNA (miRNA), cancer therapy

## Abstract

Bladder cancer (BC) is the 11th most common diagnosed cancer, and a number of factors including environmental and genetic ones participate in BC development. Metastasis of BC cells into neighboring and distant tissues significantly reduces overall survival of patients with this life-threatening disorder. Recently, studies have focused on revealing molecular pathways involved in metastasis of BC cells, and in this review, we focus on microRNAs (miRNAs) and their regulatory effect on epithelial-to-mesenchymal transition (EMT) mechanisms that can regulate metastasis. EMT is a vital process for migration of BC cells, and inhibition of this mechanism restricts invasion of BC cells. MiRNAs are endogenous non-coding RNAs with 19–24 nucleotides capable of regulating different cellular events, and EMT is one of them. In BC cells, miRNAs are able to both induce and/or inhibit EMT. For regulation of EMT, miRNAs affect different molecular pathways such as transforming growth factor-beta (TGF-β), Snail, Slug, ZEB1/2, CD44, NSBP1, which are, discussed in detail this review. Besides, miRNA/EMT axis can also be regulated by upstream mediators such as lncRNAs, circRNAs and targeted by diverse anti-tumor agents. These topics are also discussed here to reveal diverse molecular pathways involved in migration of BC cells and strategies to target them to develop effective therapeutics.

## 1. Introduction

Bladder cancer (BC) is among the most common cancers worldwide (11th most common diagnosed cancer) and its global incidence rate is higher in males compared to females (9 per 100,000 persons for males and 2.2 per 100,000 persons for females) [1,2]. According to estimates, incidence rate of BC is higher in European countries compared to the average worldwide incidence rate (19.1 for males and 4 for females) [3]. It seems that incidence and mortality of BC are different in various countries based on the risk factors, diagnostic tools and treatment availability as well as data collection strategies [4,5]. BC has a higher incidence rate in Southern and Western Europe and North America, as well in certain countries in Northern Africa or Western Asia [6].

Although etiology of BC is not completely understood, a number of factors are considered as risk factors for BC development. The most well-known risk factor is tobacco smoking which is responsible for development of 50% of BC cases [7]. Occupational exposure to agents such as aromatic amines, polycyclic aromatic hydrocarbons and chlorinated hydrocarbons can be considered as other factors involved in BC development. Genetic mutations are also involved in BC development by enhancing susceptibility into other risk factors [8,9,10]. Radiation exposure and chlorinating drinking water have been also reported to account for BC development [11,12].

BC is a heterogeneous group of tumors with up to 40 histological subgroups. Fortunately, novel therapeutics are evolving for BC therapy beyond chemotherapy, which includes immunotherapy and molecular targeted agents. Most of BC cases are urothelial carcinomas, and 10% of them are non-urothelial carcinomas [3,13]. For each of the aforementioned types of BC, there are different subtypes. For instance, urothelial carcinomas are divided into different subtypes based on histopathological profile such as conventional urothelial carcinoma, variant urothelial carcinoma and so on. The advantageous of this subtyping is of importance for precision therapy, and providing effective and separate diagnosis and treatment [14].

Although different subtypes have been developed for BC and much effort has been conducted for using chemotherapy and radiotherapy in BC therapy, the cure of BC patients remains one of the big challenges for scientists. Partly, this difficulty in treating BC is due to poor understanding of molecular profile of BC. In spite of experiments for revealing genetic factors and molecular pathways involved in BC progression and malignancy, there is still a long way, since cancer cells use dynamic and flexible molecular pathways for ensuring their proliferation and invasion. A group of these molecular signaling pathways involved in proliferation and migration of BC cells, and enhance their viability known as oncogenic pathways such as Wnt [15], STAT3 [16], Nrf2 [17], PI3K/Akt [18], ZEB [19], and oncogenic microRNAs (miRNAs) and long non-coding RNAs (lncRNAs) [20,21]. Another group of molecular pathways include those which inhibit growth and metastasis of BC cells such as PTEN [22], AMPK [23], and onco-suppressor miRNAs and lncRNAs [24,25]. These molecular pathways have been extensively studied in BC cells and there are excellent reviews about them.

One of the issues in treatment of patients with BC is the high metastatic capability of BC cells that negatively affects overall survival of patients with this life-threatening disorder, and remarkably reduces efficacy of chemotherapy and radiotherapy, since cancer cells migrate in neighboring and distant tissues, providing problems with their effective eradication [26,27,28]. Several molecular pathways such as ZEB1 [19], EZH2 [29], PI3K/Akt [30], and so on have been identified as potential factors involved in metastasis and invasion of BC cells. Epithelial-to-mesenchymal transition (EMT) is one of the most important molecular pathways that enhanced migratory ability of cancer cells [31,32]. Interestingly, EMT has been recognized as a potential downstream target of miRNAs in cancer cells [33,34,35,36,37]. In the present review, we focus on metastatic BC cells, and the role of miRNA/EMT axis. This review will provide a comprehensive discussion about the role of miRNA/EMT in malignant behavior and strategies to target this axis in BC cells.

## 2. EMT Mechanism

The first definition of EMT is transformation of epithelial cells into mesenchymal ones that have migratory features and can be accompanied by downregulation of epithelial markers such as E-cadherin, and upregulation of mesenchymal markers such as N-cadherin and vimentin [38,39]. Epithelial cells have cell-cell junctions, apico-basal polarity and low potential in migration. The epithelial cells are detected via their cell surface markers including E cadherin (as the most important and well-known factor), cytokeratins, occluding and claudins [40,41,42,43]. However, these properties are reversed in mesenchymal cells during EMT. Mesenchymal cells have front-rear polarity and potential in migration. There are surface markers by which mesenchymal cells are recognized, including N-cadherin, fibronectin and vimentin [44,45,46,47,48]. The EMT process was first observed during embryonic development. It was found that EMT is a vital mechanism for mesoderm formation and neural crest delamination. Based on dynamic property of cell identity, different mechanisms may be involved in ensuring this feature and EMT is one of them. During EMT activation, cells lose their identity and morphology to become a new one with mesenchymal characteristics [49,50]. It is worth mentioning that EMT is a reversible mechanism and its converse route is known as mesenchymal-to-epithelial transition (MET) [51].

A variety of transcriptional and epigenetic factors can contribute to regulation of EMT and MET. Although EMT is vital for physiological conditions such as organismal development, tissue healing and homeostasis, cancer cells hijack EMT to ensure their migration [52,53,54,55,56]. In order to occupy and colonize in distant tissues, cancer cells use EMT mechanism to promote their migratory ability [57,58,59,60]. It has been noted that EMT mechanism not only ensures invasion of cancer cells, but also is involved in reducing sensitivity of cancer cells into apoptosis and stimulation of chemoresistance [61,62]. EMT is not a binary state procedure and has a dynamic spectrum in which cells can have both epithelial and mesenchymal features [63,64,65,66,67,68]. Moreover, the cells undergoing EMT have more tumor-initiating potential and are resistant to apoptosis [69,70]. Furthermore, cancer cells are required to alter their metabolism in order to meet their energy needs and synthesize biomolecules such as proteins, lipids, and nucleic acids [71,72]. The most important change in metabolism of cancer cells is shifting from oxidative phosphorylation into glycolysis to meet their needs into adenosine triphosphate (ATP). This is known as Warbrug effect [73]. There is a close relationship between EMT and glucose metabolism in which glycolysis, tricarboxylic acid cycle (TCA) cycle, lipid and amino acid metabolism participate in EMT induction and stimulating invasion and migration of cancer cells [74,75].

A number of published articles have investigated the role of molecular pathways in regulation of EMT in cancer cells [41,76]. Due to space limitations, it is impossible to discuss all the molecular pathways involved in EMT regulation in cancer cells. However, we briefly discuss upstream mediators of EMT in cancer cells. Increasing evidence demonstrates that tumorigenesis and EMT can be regulated by transcription factor STAT3 in cancer cells [77,78]. In a recently published article, it was found that phosphorylation of STAT3 at tyrosine^705^ is associated with an increase in metastasis and migration of cancer cells via EMT induction. Moreover, STAT3-mediated EMT stimulation mediates resistance of cancer cells into cisplatin chemotherapy [79]. In fact, relationship between STAT3 and EMT not only is beneficial for invasion of cancer cells, but can also trigger chemoresistance. In addition to STAT3, other molecular factors have been identified as regulators of EMT. It has been reported that non-muscle myosin IIA (NMIIA) can participate in regulation of EMT in cancer cells. NMIIA can play an important role in controlling cell cytokinesis and migration. NMIIA triggers Wnt/β-catenin signaling pathway, which in turn, stimulates EMT, leading to enhance metastasis and invasion of cancer cells [80]. EMT induction is mediated via upregulation of N-cadherin and downregulation of E-cadherin [81]. LncRNAs are also able to regulate EMT in cancer cells. LncRNA FLVCR1-AS1 can also elevate migration and metastasis of ovarian cancer cells via EMT induction [82]. These studies are in agreement with the fact that different molecular pathways contribute to EMT regulation. These pathways have been examined in different cancers and their further targeting can pave the way for effective suppression of cancer metastasis [83,84,85,86].

## 3. MicroRNAs and Their Role in Cancer Metastasis

MiRs are single stranded non-coding RNAs with a length of 19–24 nucleotides [86,87]. They can negatively affect expression of target gene via binding into 3/-untranslated region (3/-UTR) [88]. MiRs have been reported to be involved in regulation of various biological mechanisms such as differentiation, apoptosis, angiogenesis, proliferation, etc. and an impairment in their expression can provide conditions for development of diverse malignancies [89,90,91,92,93]. Accumulating data demonstrates that miRs are potential upstream regulators of EMT in cancer cells. The modulatory effect of miRs on EMT can regulate the processes of metastasis and invasion of cancer cells. For example, MiR-451a is considered an onco-suppressor miR in hepatocellular carcinoma cells. This miR is able to suppress metastasis of hepatocellular carcinoma cells, and thereby interfere with migration of cancer cells by promoting inhibition of EMT [33]. Transforming growth factor-beta (TGF-β) is involved in cancer progression via EMT induction [94]. TGF-β-mediated EMT can also be targeted by miRs. It has been reported that miR-455-3p can restrict migration of cancer cells via down-regulation of TGF-β, and subsequent inhibition of EMT [95]. In addition to onco-suppressor miRs, there are miRs that can significantly increase migratory capability of cancer cells such as miR-HCC2. It has been reported that miR-HCC2 can increase the migration and metastasis of cancer cells via EMT induction [96]. In EMT induction, oncogene miRs may also affect upstream mediators of EMT. For instance, it has been documented that miR-499a-5p facilitates metastasis of lung cancer cells via upregulation of mammalian target of rapamycin (mTOR), and subsequent stimulation of EMT [35].

## 4. MicroRNAs Inhibit EMT in Bladder Cancer Cells

In the previous sections, we described EMT mechanism and its regulation by different molecular pathways with an emphasis on miRNAs. In this section, we specifically discuss the role of onco-suppressor miRNAs in inhibition of EMT in BC cells. Interestingly, expression analysis has revealed that onco-suppressor miRNAs undergo downregulation in BC cells and tissues. This occurs to ensure proliferation and migration of BC cells and is correlated with poor prognosis of patients with BC. miRNA-124-3p is an onco-suppressor miRNA and it appears that enhancing expression of this miRNA can disrupt migration of BC cells. In addition, miRNA-124-3p can inhibit EMT via E-cadherin upregulation and N-cadherin downregulation. Moreover, EMT inhibition by miRNA-124-3p relies on affecting integrin α3 (ITGA3). Increasing evidence demonstrates that ITGA3 is able to promote migration of cancer cells, and it is a downstream target of miRNAs [97,98]. In case of BC, miRNA-124-3p can target ITGA3 to suppress EMT, leading to a decrease in migration of BC cells [89]. Another study also emphasized the role of ITGA3 in metastasis of BC cells, and inhibitory effect of miRNA-328-3p on ITGA2 in suppressing BC migration [99].

### 4.1. MicroRNAs and PI3K/Akt/EMT Axis

PI3K/Akt signaling pathway can act as an inducer of metastasis in several malignancies including BC cells [100,101,102]. Anti-tumor agents such as leupaxin can suppress invasion of BC cells via PI3K/Akt downregulation [103]. miRNA-328-3p is considered as an onco-suppressor factor in BC cells. It has been found that miRNA-328-3p inhibits EMT via PI3K/Akt downregulation [99]. miRNAs are also able to affect downstream targets of PI3K/Akt signaling pathway in BC cells. B cell-specific Moloney murine leukemia virus integration site 1 (BMI1) is suggested to undergo upregulation in different cancers. BMI1 overexpression ensures tumor sphere formation of cancer cells, and its stability is a positive factor for tumorigenesis [104,105]. MiR-15 as an onco-suppressor factor has been reported to inhibit EMT in BC cells. It appears that miRNA-15 inhibits PI3K/Akt signaling pathway to suppress BMI1 expression, leading to EMT inhibition and decreased invasion of BC cells [106].

### 4.2. MicroRNAs and CARMA3/EMT Axis

An extensive amount of research has focused on various factors involved in metastasis of BC cells and a considerable role of EMT in malignant behavior and metastasis of these malignant cells has been established [107,108]. Interestingly, CARD-containing MAGUK 3 (CARMA3) is a scaffold with capability of stimulation of nuclear factor-kappa B (NF-κB) signaling pathway and enhancing tumor growth [109,110,111,112]. downregulation of CARMA3 is of interest in suppressing metastasis of BC cells, since CARMA3 is able to induce matrix metalloproteinase-2 (MMP-2), MMP-9 and c-Myc expression that are involved in the migration of BC cells. Besides, CARMA3 can reduce the levels of E-cadherin, whereas it increases β-catenin levels [113]. Moreover, miRNA-24 has gained much attention as a therapeutic target in BC therapy and it has been noted that that miRNA-24 can down-regulates the expression of CARMA3 to suppress EMT via increasing E-cadherin levels, and decreasing N-cadherin, vimentin and MMP-9 levels [114].

### 4.3. MicroRNAs and Wnt/EMT Axis

The Wnt/β-catenin signaling pathway has been under attention in recent years due to its role in tumorigenesis [115,116,117,118,119]. The tumor-promoting role of Wnt signaling pathway has been investigated in different cancers, including BC [120,121]. It has been found that Wnt signaling can act as an upstream mediator of EMT, so that after activation of Wnt signaling pathway by certain ligands of Wnt family, GSK-3β activity can be inhibited to facilitate the nuclear translocation of β-catenin. Thereafter, various downstream targets with stimulatory effect on proliferation and migration of cancer cells are activated [122,123,124,125]. EMT is a downstream effectt of Wnt activation in cancer progression [86,126]. miRNAs are able to regulate Wnt/EMT axis in BC cells. miRNA-3619-5p as an onco-suppressor factor in BC cells, is able to inhibit metastasis of BC cells. Moreover, examination of molecular pathways shows that miRNA-3619-5p can inhibit Wnt signaling pathway via suppressing nuclear translocation of β-catenin. As a consequence, a decrease occurs in EMT and mesenchymal markers causing an inhibition in invasion of BC cells [127]. Moreover, it has been demonstrated that Wnt7a is capable of induction of Wnt signaling pathway that in turn, enhances β-catenin levels to stimulate EMT, leading to increased migration and invasion of BC cells. miRNA-370-3p can suppress Wnt-mediated EMT via inhibition of EMT [115], thus making it an important therapeutic target for BC therapy.

### 4.4. MicroRNAs and EMT-Inducing Transcription Factors

**Figure 1:**
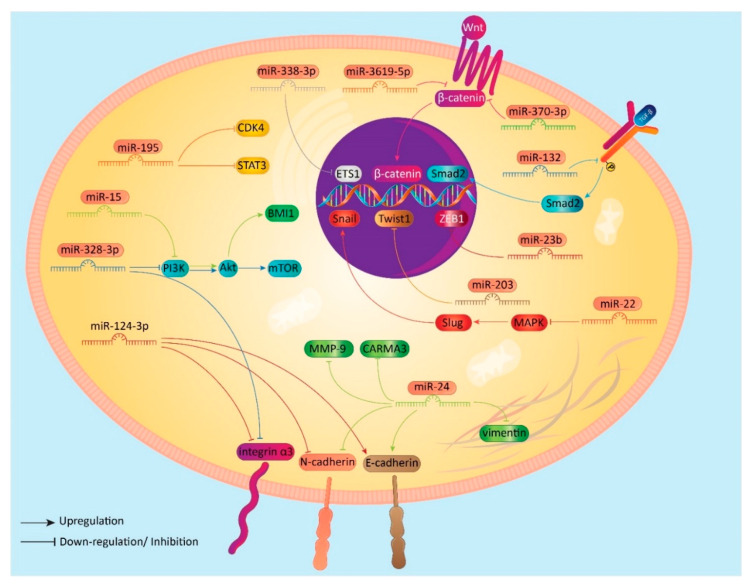
Inhibition of EMT in BC cells by miRNAs.

TGF-β is another signaling pathway that plays a vital role in cancer malignancy [128]. Briefly, TGF-β induces the formation of Smad complex that can translocate into the nucleus and affect target genes [129,130]. TGF-β can promote migration and metastasis of cancer cells via EMT induction [131,132]. In BC cells, TGF-β1 induces nuclear translocation of Smad2, which in turn, stimulates EMT and can enhance BC migration. miRNA-132 inhibits TGF-β1/Smad2/EMT axis in suppressing BC invasion [133]. In addition to TGF-β, ZEB1 can also induce EMT. Both TGF-β and ZEB1 belong to EMT-inducing transcription factors (EMT-TFs). Similar to TGF-β, ZEB1 is able to stimulate EMT in promoting invasion and metastasis of cancer cells [134,135]. In BC cells, miRNAs are able to affect ZEB1 expression. For instance, miRNA-23b interferes with migration of BC cells into distant tissues by EMT inhibition via downregulation of ZEB1 [136]. Twist1 is another member of EMT-TFs. Twist1 is an enhancer of EMT in cancer cells and has been linked with invasion and migration [137,138]. miRNA-203 has demonstrated onco-suppressor role in BC cells by causing a downregulation of Twist1. Decreased expression of Twist1 is associated with a diminution in EMT and limited migration of BC cells [139]. Slug can also be affected by miRNAs in BC cells, which is able to induce EMT, and its activity can be regulated by MAPK [140,141]. MAPK/Slug/EMT axis is of importance in BC cells, and miRNAs have demonstrated great potential in its regulation. On the contrary, miRNA-22 as an onco-suppressor is able to inhibit MAPK signaling pathway [142,143]. A recently published study has examined the effect of miRNA-22 on MAPK/Slug/EMT axis in BC cells. It was found that miRNA-22 can bind to 3/UTR of MAPK to inhibit its activity. Consequently, MAPK-induced Slug expression can be reduced and a decrease occurs in the levels of vimentin, as a marker of mesenchymal cells. These effects result in inhibition of EMT in BC cells. Furthermore, miRNA-22 can suppress Snail in causing EMT inhibition in BC cells [144]. These studies are in agreement with the fact that miRNAs are able to target EMT-TFs in suppressing metastasis of BC cells. In addition, because of substantial capability of miRNAs in forming feedback loops among divesre oncogenic molecular pathways, it appears that anti-tumor agents or genetic tools applied for targeting aforementioned signaling pathways may not be effective enough to overcome cancer metastasis and EMT [145,146,147,148]. In fact, eradication of BC cells, and suppressing their migration may depend on using a combination of anti-tumor agents (poly-chemotherapy) capable of targeting various molecular pathways or using genetic tools for silencing various downstream targets of miRNAs that are involved in regulating EMT (Table 1, Figure 1).

### 4.5. miRNA-200 Family and EMT

A well-known family of miRNAs that are capable of EMT regulation in different cancers is miRNA-200 family [165,166]. The EMT inhibition by miRNA-200 is a gateway for enhancing efficacy of chemotherapy [167]. ZEB proteins that can act as critical enhancers of EMT are often downregulated by miRNA-200 to suppress cancer migration (EMT) [168]. Noteworthy, two studies have evaluated role of miRNA-200 in affecting BC metastasis via EMT regulation. These studies are in accordance with our aforementioned discussions and show that EMT regulation by miRNA-200 leads to enhanced efficacy of chemotherapy and migration inhibition. EMT induction by ZEB proteins (ZEB1 and ZEB2) is a factor involved in resistance of BC cells into chemotherapy. Stable expression of miRNA-200 leads to inhibition of ZEB1 and ZEB2 that can lead to an increased levels of E-cadherin, thus causing a reduced migratory ability of BC cells [160]. This study highlights the fact that cancer may acquire chemoresistance, when they demonstrate malignant behaviors such as invasion. Therefore, EMT acts as a critical factor in cancer metastasis, and signaling pathways regulating this mechanism need to be identified in detail, and miRNAs are possibly one of them. Another study investigated the relationship between miRNA-200 and EMT in BC migration, while previous study demonstrated involvement of miRNA-200/EMT axis in BC chemoresistance. It has been reported that miRNA-200 can reduce expression of BMI and E2F3 as inducers of BC metastasis that results in EMT inhibition via E-cadherin upregulation [161]. Further studies can focus on identifying more downstream targets of miRNA-200 and how they can regulate both EMT and metastasis of BC cells.

### 4.6. Other microRNAs

In addition, clinical studies have shown that miRNA-195 undergoes downregulation in patients with BC, and is associated with undesirable prognosis [169]. This miRNA is able to suppress proliferation and progression of BC cells via STAT3 inhibition [170]. Moreover, a downregulation of miRNA-195 by lncRNA UCA1 can promote mitochondrial function and growth of BC cells [171]. miRNA-195 is also able to inhibit EMT in BC cells via CDK4 inhibition [172]. In addition, studies have demonstrated that different molecular pathways ensure progression and migration of BC cells. For example, E26 transformation specific-1 (ETS1) is an oncogene factor in BC cells, and its induction can elevate migration of BC cells [173,174]. There is a negative relationship between miRNA-338-3p and ETS1 in BC cells. Moreover, by causing a downregulation of ETS1, miRNA-338-3p can abrogate EMT in BC cells to suppress migration of BC cells [175].

## 5. MicroRNAs can Induce EMT in BC Cells

### 5.1. MicroRNAs and Wnt/EMT Axis

In previous section, we have highlighted that Wnt signaling pathway contributes to stimulation of EMT via β-catenin’s nuclear translocation. It was revealed that miRNA-3619-5p acts as an onco-suppressor, targets nuclear translocation of β-catenin. However, it has been reported that miRNAs can also target GSK-3β activity. miRNA-135a has demonstrated an oncogene role in BC cells. It appears that miRNA-135a induces EMT mechanism to promote metastasis of BC cells. Investigation of molecular pathways showed that miRNA-135a can induce Wnt/ β-catenin signaling pathway via inhibiting GSK-3β activity. Therefater, β-catenin undergoes nuclear translocation that activates EMT, leading to enhanced migratory ability of BC cells [176]. miRNA-135a/Wnt/EMT can therefore be targeted in further studies to inhibit the migration of BC cells.

### 5.2. MicroRNAs and EMT-Inducing Transcription Factors

Although onco-suppressor miRNAs reduce expression of EMT-TFs to suppress EMT in BC cells, there are oncogenic miRNAs capable of enhancing expression of EMT-TFs in EMT induction. TGF-β1 is able to induce EMT in BC cells and miRNA-96 as an oncogene factor upregulating the expression of TGF-β1 to stimulate EMT in BC cells and thus promoting their migration and invasion [177]. Stathmin 1 (STMN1) is a microtubule-destabilizing protein that plays a vital role in mitosis and cell cycle progression [178,179]. STMN1 is considered as an oncogenic protein, its induction enhances invasion and metastasis of cancer cells [180,181]. It has been reported that miRNA-221 can induce EMT in BC cells via induction of TGF-β. The stimulatory effect of miRNA-221 on TGF-β-mediated EMT relies primarily on inhibition of STMN1 [182].

### 5.3. MicroRNAs and EGR1/EMT Axis

In addition, early growth response gene 1 (EGR1) acts as a double-edged sword in cancer by displaying both tumor promoting and suppressing roles. EGR1 can reduce apoptosis of cancer cells and promote their migration and angiogenesis [183,184]. However, there are studies showing anti-tumor activity of EGR1 and its role in suppressing metastasis of cancer cells [185]. Moreover, miRNA-301b and EGR1 can act in conjunction to regulate EMT in BC cells. It was found that miRNA-301b has high expression in BC cells, while EGR1 undergoes downregulation in these malignant cells. Investigation of molecular pathways revealed that miRNA-301b can induce EMT in BC cells via EGR1 downregulation [186].

### 5.4. Other microRNAs

The difficulty in inhibiting metastasis of BC cells is due to involvement of different and complicated molecular pathways in progression of BC cells. RhoB is key member of Rho family and a GTP-binding protein. Increasing evidence demonstrates that RhoB undergoes downregulation in cancer cells and is correlated with poor prognosis [187,188]. RhoB has also been found to inhibit metastasis via EMT downregulation [189]. Based on inhibitory effect of RhoB on EMT, its activity and expression should be diminished by oncogenic miRNAs. Indeed, it was reported that miRNA-19a can promote metastasis and invasion of BC cells by EMT induction via reducing RhoB levels [190].

**Figure 2:**
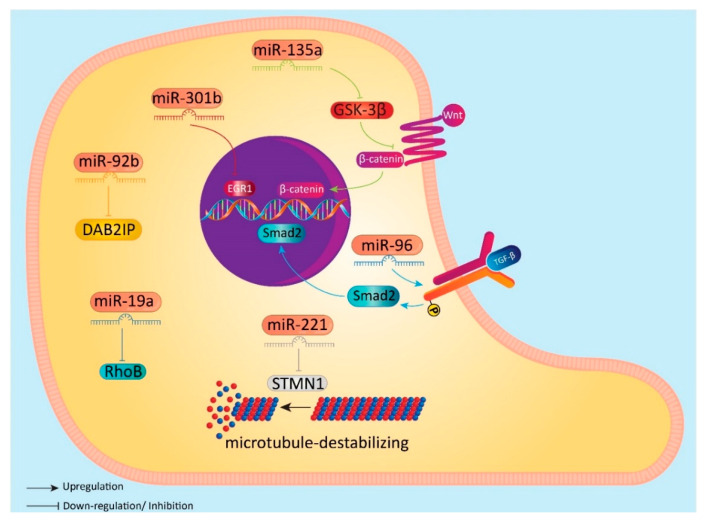
Induction of EMT by miRNAs in BC cells.

DAB2IP is a member of RAS-GTPase-activating protein family and its expression has been found to be downregulated in cancer cells [191]. It has been shown that inhibition of DAB2IP in BC cells can mediate both chemoresistance and metastasis [192,193]. Therefore, targeting DAB2IP is of interest for minimizing the migration of BC cells. In order to ensure invasion of BC cells, miRNA-92b can affects DAB2IP effectively. In fact, by causing an inhibition of DAB2IP, miRNA-92b induces EMT, leading to enhanced migration and invasion of BC cells [194]. These studies clearly establish that miRNAs are able to induce EMT in BC cells. However, most of the studies have focused on onco-suppressor miRNAs, and their capability in inhibition of EMT in BC cells. In addition, other studies have also paid attention into regulation of miRNA/EMT axis by other important molecular signaling pathways. Hence, more studies are needed to identify oncogenic miRNAs, which can induce EMT in BC cells to target them effectively for suppressing migration and metastasis of BC cells (Figure 2).

## 6. Regulation of mciroRNA/EMT in Bladder Cancer Cells

### 6.1. LncRNAs as Main Regulators

Although miRNAs are potential upstream modulators of EMT in BC cells, and according to previously reported findings, they can target EMT via affecting different molecular pathways. In addition, there are studies showing that several important molecular pathways can also influence miRNAs. This demonstrates the complexity of molecular pathways involved in regulating EMT in BC cells. Similar to miRNAs, lncRNAs are key members of non-coding RNA family that can participate in regulation of vital biological mechanisms such as cell proliferation and cell differentiation [183,195]. A number of studies have shown a significant role of lncRNAs in cancer cells, both as an oncogene or onco-suppressor [196,197]. Both miRNAs and lncRNAs can control the regulation of protein-coding genes [198]. LncRNAs are able to suppress interaction of miRNAs with their target via acting as molecular decoys and sequestering miRNAs [199,200]. Notably, miRNAs are downstream targets of lncRNAs in BC cells [201,202]. This dual relationship can be studied to draw a unique axis and potential therapeutic targeting in future studies. miRNAs are capaable to affect EMT-TFs involved in regulation of EMT in BC cells. LncRNA SNHG6 is an oncogene factor that can promote proliferation of cancer cells, and its downregulation can restrict malignant behavior of cancer cells [138]. Accumulating data demonstrates that SNHG6 can induce EMT in cancer cells via targeting EMT-TFs such as TGF-β and ZEB1 [203], making it an efficient target in suppressing metastasis of cancer cells.

For instance, SNHG6/miRNA-125b/EMT has been implicated in the migration of BC cells. It has been reported that SNHG6 can down-regulate the expression of miR-125b as an onco-suppressing factor. Therefore, an increase occurs in expression of Snail1/2 and ZEB1 to induce EMT, leading to an enhanced metastasis of BC cells [204]. LncRNA H19 is an enhancer of growth and migration in BC cells. Increasing evidence demonstrates that H19 is able to enhance metastasis of BC cells by reducing E-cadherin levels via EZH2 induction [205]. This inhibitory effect of lncRNA H19 on E-cadherin levels can provide condition for migration and invasion of BC cells and undesirable prognosis [206]. An experiment has examined the relationship between lncRNA H19 and miRNA-29b-3p in regulation of EMT in BC cells. This study found that LncRNA H19 can function as an inhibitor of miRNA-29b-3p by sponging. Consequently, an increase occurs in expression of DNMT3B, as a downstream target of miRNA-29b-3p. This provides condition for induction of EMT by H19/miRNA-29b-3p/DNMT3B axis. Inhibition of lncRNA H19 leads to inhibition of EMT and stimulation of MET to suppress metastasis of BC cells [207]. It is worth highlighting that lncRNAs may indirectly affect EMT and metastasis of BC cells. The final aim of targeting miRNAs by lncRNAs in BC cells is to modulate downstream targets of miRNAs and regulate EMT mechanism. For instance, lncRNA LINC00612 is able to induce PHF14 expression via miRNA-590 inhibition, leading to EMT induction and metastasis of BC cells [208]. These studies are in agreement with the fact that lncRNAs can substantially affect miRNA/EMT axis in BC cells. However, more studies are needed to examine the relationship between lncRNAs and miRNA/EMT in BC cells to unravel this intriguing association.

### 6.2. CircRNAs as Main Regulators

Circular RNAs (circRNAs) are another member of non-coding RNAs capable of regulating miRNAs in cancer cells [190,209]. CircRNAs can be divided into onco-suppressor and oncogenic in BC. They are able to target different molecular pathways in BC cells and miRNAs are their major downstream targets [210,211]. In BC cells, circRNA circPICALM functions as an onco-suppressor factor via causing downregulation of miRNA-1265. The expression of this circRNA undergoes downregulation in BC cells and tissues and has been correlated with poor prognosis. In addition, analysis of molecular pathways has demonstrated that circPICALM can reduce the expression of miRNA-1265. STEAP4, as a downstream target miRNA-1265 undergoes upregulation, which in turn, can inhibit phosphorylation of FAK at Y397, resulting in inhibition of EMT and metastasis of BC cells [212]. In addition to miRNA-1265, it appears that miRNA-221 may be affected by circRNAs in BC cells. For instance, miRNA-221 is an oncogene factor that can reduce the number of cancer cells undergoes apoptosis to ensure proliferation and survival of BC cells [213]. Besides, miRNA-221 has also demonstrated immunosuppressor activity and can facilitate immune evasion of BC cells [214]. So, targeting miRNA-221 is of importance in suppressing malignant behavior of BC cells. Another example is that of CircMTO1 that acts as an onco-suppressor to inhibit miRNA-221 in BC cells. It appears that inhibition of miRNA-221 by circMTO1 may be related to a decrease in migration of BC cells. This is due to inhibition of miRNA-221 and enhancement of E-cadherin/N-cadherin ratio, thereby leading to EMT and metastasis inhibition of BC cells [183]. It is also noteworthy that circRNAs are able to indirectly affect EMT-TFs in BC cells by targeting miRNAs. As it was mentioned earlier, TGF-β can act as a promoter of EMT in cancer cells and suppressing TGF-β ca be promising strategy in metastasis inhibition. Moreover, CircRIP2 can function as an oncogenic factor in BC cells that can trigger EMT to ensure metastasis and malignant behavior of tumor cells. Interestingly, investigation of molecular pathways indicated that circRIP2 could reduce the expression of miRNA-1305 that in turn, can inhibit TGF-β2 and its downstream target, Smad3. This eventually can lead to inhibition of EMT and decreased invasion of BC cells [215].

### 6.3. Anti-Tumor Compounds as Main Regulators

**Figure 3:**
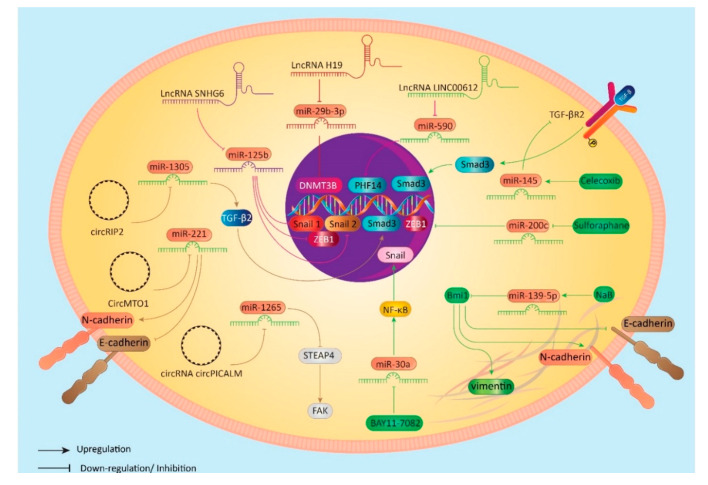
Regulation of miRNA/EMT axis in BC cells.

It is worth highlighting that pharmacologically active anti-tumor drugs are also able to target miRNAs for cancer therapy. Sulforaphane is a plant-derived natural compound exclusively found in cruciferous vegetables [216]. It has demonstrated high anti-tumor activity by induction of apoptosis and regulation of miRNAs, that can sensitize cancer cells to chemotherapy [217]. In BC cells, administration of sulforaphane has been suggested to be an ideal candidate for suppressing invasion and migration of cancer cells. In order to inhibit EMT in BC cells, sulforaphane can effectively target miRNA-200c/ZEB1 axis. miRNA-200c is an onco-suppressor factor and its upregulation by sulforaphane can lead to an inhibition of ZEB1 expression. This in turn can effectively suppress EMT and metastasis of BC cells [218]. Chronic inflammation acts as a positive factor for growth and malignancy of cancers, including BC [219,220]. Tumor-associated macrophages (TAMs) are key members of tumor microenvironment and their abundancy has been correlated with metastasis of cancer cells, and unfavorable prognosis [221,222]. BAY11-7082 as an inhibitor of oncogenic NF-κB signaling pathway [223,224,225,226] is able to inhibit stimulatory action of TAMs on EMT in BC cells. Normally, TAMs upregulate expression of miRNA-30a to induce NF-κB/Snail signaling pathway, leading to EMT and invasion of BC cells. BAY11-7082 can decrease expression of miRNA-30a to disrupt NF-βB/Snail axis, thereby restricting invasion of BC cells via inhibition of EMT [227]. Sodium butyrate (NaB) is a histone deacetylase inhibitor that is extensively applied in cancer therapy for inhibition of migration and induction of apoptosis via mitochondrial dysfunction [228,229,230,231]. Interestingly, administration of NaB has been found to be important in suppressing EMT in BC cells. It appears that NaB can enhance expression of miRNA-139-5p that in turn may reduce Bmi1 expression. Thus, miRNA-139-5p/Bmi1 axis cans lead to an increase in E-cadherin levels and a decrease in Snail, N-cadherin and vimentin levels to inhibit EMT in BC cells [232]. Celecoxib is another anti-tumor agent capable of suppressing cancer proliferation via induction of oxidative stress [233]. Celecoxib is able to suppress metastasis of cancer cells via EMT inhibition [234]. A recent study has shown that celecoxib is able to target miRNAs in regulation of EMT in BC cells. Administration of celecoxib enhanced the expression of miRNA-145 that in turn, inhibited TGFβR2. This leads to an inhibition of Smad3 and a decrease in migration and metastasis of BC cells via suppressing EMT [235]. These studies are in agreement with the fact that anti-tumor compounds can affect miRNAs in regulation of EMT in BC cells and more studies will be needed to discover novel anti-tumor drugs with modulatory impact on miRNA/EMT axis in BC cells. To date, a variety of studies have examined regulation of miRNA/EMT axis in BC cells. LncRNAs are major upstream regulators of miRNA/EMT axis in BC cells and experiments have extensively investigated their relationship with metastasis of BC cells. The p53 and KCNQ1OT1 are other upstream mediators capable of regulating miRNA/EMT axis in BC cells [236,237]. Overall, identification of these signaling pathways will enable us to target them in further studies and effectively inhibit metastasis of BC cells (Table 2, Figure 3).

## 7. Conclusions and Future Directions

Based on the role of miRNAs in regulation of different cellular events, a disturbance in their expression leads to development of pathological events, particularly cancer. miRNAs have been under attention for regulating both proliferation and invasion of BC cells, and to date, a substantial number of studies have identified oncogene and onco-suppressor miRNAs in BC cells [22,25,211,245,246,247,248]. On the other hand, EMT can act as a downstream target of miRNAs in BC. EMT considerably enhances metastasis and migration of BC cells, and its targeting is of importance in suppressing invasion of BC cells and thereby providing desirable prognosis. In the present review, we have provided a comprehensive review about role of miRNAs in regulation of EMT in BC cells. We have divided discussion section into three parts including onco-suppressor miRNAs in EMT inhibition, oncogene miRNAs in EMT induction, and regulation of miRNAs by other molecular pathways such as lncRNAs, circRNAs, etc. Each of the above-mentioned section has investigated a unique signaling pathway in which miRNAs can function as key players regulating tumorigenesis. Targeting these molecular pathways can be considered as an efficient therapeutic strategy in suppressing EMT in BC cells and elevating overall survival of patients with BC.

## Figures and Tables

**Table 1:** MicroRNAs inhibit EMT in BC cells.

MicroRNA	Downstream Target	Cell Line	Major Outcomes	Refs
miRNA-370	SOX12	Immortalized bladder cell line SV-HUC-1 (ATCC^®^ CRL-9520™) and the human BC cell lines 5637 (ATCC^®^ HTB-9™) and J82 (ATCC^®^ HTB-1™)	miRNA-370 inhibits EMT via SOX12 downregulation, leading to a decreased metastasis of cancer cells	[149]
miRNA-34a	CD44	Human bladder cancer cell lines (5637, T24, HT-1376, J82, SCABER and EJ)	Suppressing EMT via CD44 inhibition	[150]
miRNA-613	SphK1	Bladder cancer cell lines (J82, T24, UMUC3 and 5637) and a normal bladder cell line (SV-HUC-1)	Inhibition of SphK1 and reduced metastasis and EMT	[151]
miRNA-186	NSBP1	Human bladder cancer cell lines (J82, HT1376, RT4, T24 and TCCSUP) and immortalized human bladder epithelium (HCV29) cells	downregulation of NSBP1 and suppressed metastasis of cancer cells	[152]
miRNA-125a-5p	FUT4	Bladder cancer cell lines (J82, T24, 5637 and BIU-87) and im-mortalized bladder cell line (SV-HUC-1)	miRNA-125a-5p inhibits invasion and EMT in BC cells via FUT4 downregulation	[153]
miRNA-203a	SIX4	SV-HUC-1 human uro-epithelial cells and the bladder cancer cell lines T24, EJ, J8 and 5637	There is a negative relationship between miRNA-203a and SIX4. This miRNA inhibits EMT via SIX4 downregulation	[154]
miRNA-22	E2F3	Human bladder cancer cell lines (5637 and T24)	Preventing expression of E2F3 and suppressed BC metastasis and EMT	[155]
miRNA-454-3p miRNA-374b-5p	ZEB2	SV-HUC, TCC, 253J, 5637, J82, T24, EJ, HEK-293T cells	Suppressing EMT via ZEB2 downregulation	[156]
miRNA-451	-	T24, 5637 and J28 bladder cancer cell lines	Inhibiting EMT via E-cadherin upregulation, and N-cadherin and vimentin downregulation	[157]
miRNA-199a-5p	CCR7	Human bladder cancer T24 cell line and human normal bladder epithelial cell line SV-HUC-1	Interfering with metastasis of cancer cells by downregulation of CCR7, and subsequent inhibition of EMT	[158]
miRNA-7-5p	Gli3	TCC, 253J, 5637, T24, EJ, J82 (BCa cell lines) and SV-HUC (human bladder epithelium immortalized cell)	miRNA-7-5p reduces expression of Gli3 as a member of Hedgehog signaling pathway to suppress EMT	[159]
miRNA-200	-	UMUC series of urothelial carcinomas and 253J BV cells	miRNA-200 enhances E-cadherin levels, and reduces ZEB1 and ZEB2 levels to inhibit EMT, leading to a diminution in metastasis and enhanced sensitivity into chemotherapy	[160]
miRNA-200c	BMI1 E2F3	Human bladder cancer cell lines (UMUC-3 and T24)	Suppressing EMT and increasing E-cadherin levels via inhibition of BMI1 and E2F3	[161]
miRNA-485-5p	HMGA2	Human bladder cancer cell lines (SW780, T24, HT1376 and HT5637) and human bladder epithelial cell lines HU609 and HEK293 cell	Disrupting invasion of cancer cells by EMT inhibition via HMGA2 downregulation	[162]
miRNA-429	ZEB1/2 β-catenin	Human UCC cell lines, T24	Enhancing E-cadherin level via ZEB1/2 downregulation and subsequent inhibition of EMT Suppressing nuclear translocation of β-catenin and its interaction with TCF/LEF1, resulting in EMT inhibition	[163]
miRNA-381-3p	CCNA2	T24, UM-UC3, and 5637 human BCa cell lines	Inhibition of EMT via CCNA2 downregulation	[164]

**Table 2:** Regulation of microRNA/EMT axis in BC cells.

Upstream Regulator	MicroRNA	Downstream Target	Cell Line	Major Outcomes	Refs
LncRNA DANCR	miRNA-149	MSI2	Bladder cancer 5637, SW780, UM-UC-3, T24 and SV-HUC-1 cells	DANCR enhanced metastasis and invasion of cancer cells by downregulation of miRNA-149, and subsequent activation of MSI2, resulting in EMT	[238]
LncRNA ARSR	miRNA-129-5p	SOX4	RT4 and 5637 cells	ARSR reduces expression of miRNA-129-5p via sponging to upregulate SOX4, leading to EMT	[239]
LncRNA XIST	miRNA-200c	-	Human bladder cancer cell lines 5637 and T24	Stimulation of EMT by downregulation of miRNA-200c	[240]
LncRNA UCA1	miRNA-143	HMGB1	Human bladder cancer cell lines (T24, 5637, J82, RT4 and HT1376)	UCA1 induces expression of HMGB1 via miRNA-143 downregulation, leading to EMT	[241]
LncRNA UCA1	miRNA-145	ZEB1/2	Human bladder cancer cells 5637, T24, and UMUC2	Stimulation of EMT by downregulation of miRNA-145 and subsequent activation of ZEB1/2	[242]
LncRNA AC114812.8	miRNA-371b-5p	FUT4	Human BC cell lines T24, UM-UC-3, J82, and 5637, and the human immortalized normal urinary epithelial cell line SV-HUC-1	Sponging of miiR-371b-5p by AC114812.8 leads to induction of FUT4, and EMT	[243]
LncRNA TUG1	miRNA-145	-	SV-HUC-1 cells	TUG1 induces EMT via downregulation of miRNA-145	[244]

## Abbreviations

BC	bladder cancer
miRNA	microRNA
lncRNA	long non-coding RNA
EMT	epithelial-to-mesenchymal transition
MET	mesenchymal-to-epithelial transition
NMIIA	non-muscle myosin IIA
3^/^-UTR	3^/^-untranslated region
TGF-β	transforming growth factor-β
mTOR	mammalian target of rapamycin
EMT-TFs	EMT-promoting transcription factors
ITGA3	integrin α3
ETS1	E26 transformatin specific-1
CARMA3	CARD-cotnaining MAGUK 3
NF-βB	nuclear factor-kappaB
MMP-2	matrix metalloproteinase-2
STMN1	stathmin 1
EGR1	early growth response gene 1
circRNAs	circular RNAs
TAMs	tumor-associated macrophages
NaB	sodium butyrate
